# Can We Disrupt the Sensing of Honey Bees by the Bee Parasite *Varroa destructor*?

**DOI:** 10.1371/journal.pone.0106889

**Published:** 2014-09-16

**Authors:** Nurit Eliash, Nitin Kumar Singh, Yosef Kamer, Govardhana Reddy Pinnelli, Erika Plettner, Victoria Soroker

**Affiliations:** 1 Institute of Plant Protection, Agricultural Research Organization, The Volcani Center, Bet Dagan, Israel; 2 Department of Chemistry, Simon Fraser University, Burnaby, B. C., Canada; University of North Carolina, Greensboro, United States of America

## Abstract

**Background:**

The ectoparasitic mite, *Varroa destructor*, is considered to be one of the most significant threats to apiculture around the world. Chemical cues are known to play a significant role in the host-finding behavior of *Varroa*. The mites distinguish between bees from different task groups, and prefer nurses over foragers. We examined the possibility of disrupting the *Varroa* – honey bee interaction by targeting the mite's olfactory system. In particular, we examined the effect of volatile compounds, ethers of *cis* 5-(2′-hydroxyethyl) cyclopent-2-en-1-ol or of dihydroquinone, resorcinol or catechol. We tested the effect of these compounds on the *Varroa* chemosensory organ by electrophysiology and on behavior in a choice bioassay. The electrophysiological studies were conducted on the isolated foreleg. In the behavioral bioassay, the mite's preference between a nurse and a forager bee was evaluated.

**Principal findings:**

We found that in the presence of some compounds, the response of the Varroa chemosensory organ to honey bee headspace volatiles significantly decreased. This effect was dose dependent and, for some of the compounds, long lasting (>1 min). Furthermore, disruption of the *Varroa* volatile detection was accompanied by a reversal of the mite's preference from a nurse to a forager bee. Long-term inhibition of the electrophysiological responses of mites to the tested compounds was a good predictor for an alteration in the mite's host preference.

**Conclusions:**

These data indicate the potential of the selected compounds to disrupt the *Varroa* - honey bee associations, thus opening new avenues for *Varroa* control.

## Introduction

Chemical cues play an important role in host-parasite interactions. Parasites often eavesdrop on their host's chemical signals, and rely on these signals for host detection and choice [1]. Parasitism of social insects is an especially complex case, as numerous chemical signals (semiochemicals) are crucial for the function of the society, including its protection from inquilines. Although semiochemicals are well known tools in pest management, in the enclosed and crowded environment of the colony, the proximity between the host and parasites presents an obstacle when one tries to confront the parasite, without damaging the host. Such a challenging situation is well known in colonies of the European honey bee *Apis mellifera* infected by the obligatory ectoparasitic mite, *Varroa destructor*. An expansion of the mite's host range from its original host (the Eastern honey bee *Apis cerana*) to a new host, *A. mellifera*, has led to an unbalanced host-parasite relationship and a devastating damage to *A. mellifera*-based apiculture [2]. Today, *Varroa* is considered one of the most significant threats to world apiculture.

Mites attach themselves to nurse bees and feed on the hemolymph of their host bee. This stress shortens the bee's life span, decreases its weight, the lifetime flight duration and non associative learning abilities [3]–[5]. *Varroa* mites also serve as an active vector of pathogenic viruses, which have become more abundant and virulent since the emergence of the mite [6], [7]. In addition, the mere parasitism by the mite weakens the bee's immune system and makes it more vulnerable to other secondary pathogens [8]. The life cycle of *Varroa* can be generally divided into two main phases: a phoretic phase, in which the *Varroa* is parasitizing an adult bee, and a reproductive phase, in which the *Varroa* is reproducing within a sealed brood cell. Between these phases the mites are shortly present on the surface of the comb. The entrance of the fertilized *Varroa* female into a brood cell is synchronized with the developmental stage of the larvae and occurs just before the cell is capped [9].

Studies have indicated that chemical cues play a major role in host finding and preference of *Varroa*. In laboratory bioassays *Varroa* has been shown to discriminate between bees from different task groups and to prefer a nurse over a forager [10], [11]. The host preference is apparently based on both low volatility compounds, such as cuticular hydrocarbons [12], and on volatile compounds emitted by the honey bees and their environment (such as larval food and brood pheromone) [13]–[15]. Despite much progress in the identification of host olfactory cues guiding *Varroa*, neither effective attractants nor repellents have been found so far. In view of limited success in exploiting hive semiochemicals in *Varroa* control, the use of synthetic disruptive compounds can be another approach to confront the mite [16]. Recently, a library of volatile compounds was developed for the disruption of chemical detection by the gypsy moths' antenna [17], [18]. These chemicals apparently interact with the pheromone binding proteins and/or other components of the olfactory system and take an effect only in the presence of a positive chemical stimulus [17], [19], [20]. As the chemical environment of the hive is rich in volatiles, it was interesting to explore the effect of these compounds on host detection and behavior of the *Varroa* mite. The ideal situation would be to confuse *Varroa* without disrupting honey bee communication in the colony.

The general location of the olfactory organ differs in mites and insects. In honey bees, like in all insects, the antennae are the major olfactory organ, whereas mites lack antennae and, therefore, the olfactory organ of *Varroa* is located on the distal part of its forelegs, analogous to the sensory pit (Haller's organ) found in ticks [21], [22]. Although chemosensory sensilla in the mite's sensory pit appear similar to those described in insects, not much is known about the mechanism behind odorant detection in mites in general and *Varroa* in particular. Only a few attempts of electrophysiological recordings from the *Varroa* foreleg have been mentioned in the literature [22], [23], [24] and recently by Eliash [25]. Furthermore, the response of the organ to honey bee volatiles had not been confirmed prior to this study. In the current study we have further established the ability to measure the response of the *Varroa* foreleg to host (honey bee) volatiles. Subsequently, we evaluated the effect of the potentially disruptive compounds on this response, as well as on the mite's ability to distinguish between two host types (a nurse and a forager bee).

## Materials and Methods

No human or animal subjects were used in this research. Bees were kept at the Agricultural Research Organization using standard apicultural methods.

Two methods were implemented to assess the effect of potential disrupting compounds. Electrophysiology was used to assess the effect of the compounds on the sensitivity of the *Varroa* chemosensory organ to honey bee volatiles. Using a behavioral bioassay, we examined if the compounds alter the *Varroa* preference for a nurse over a forager bee.

### Biological material

Honey bee colonies (*A. mellifera liguistica*) were maintained at an experimental apiary at Beit Dagan, ARO the Volcani Center, Israel. The experimental hives were maintained without any treatment against *Varroa*, but they received seasonal sugar feeding and Fumagilin treatment against Nosema.

Female adult *Varroa* mites were regularly collected from a tray under a screen net at the bottom of the hive. Even though mites from the bottom of the hive could be of diverse age and physiological conditions we found this factor insignificant for the mites' host selection. In our preliminary data the behavior of mites collected from trays did not differ from that of mites from sealed brood (Eliash, unpublished). All mites were kept on a moist filter paper at room temperature up to 4 hours prior to the experiments. Adult honey bees of two task groups (nurse and foragers) were collected for the experiments. Honey bees observed leaning into brood cells were regarded as nurse bees whereas pollen foragers, carrying pollen loads, were collected from the entrance of the hive according to Kather et al. [26]. The bees were killed by freezing at −20°C, for 1 hour. Prior to a behavioral bioassay, the pollen loads were thoroughly removed from forager bees by using forceps under stereo microscope (Olympus DF PLAPO 1XPF JAPAN). Nurses were used as taken from the hive.

### Chemical compounds

**Figure 1:**
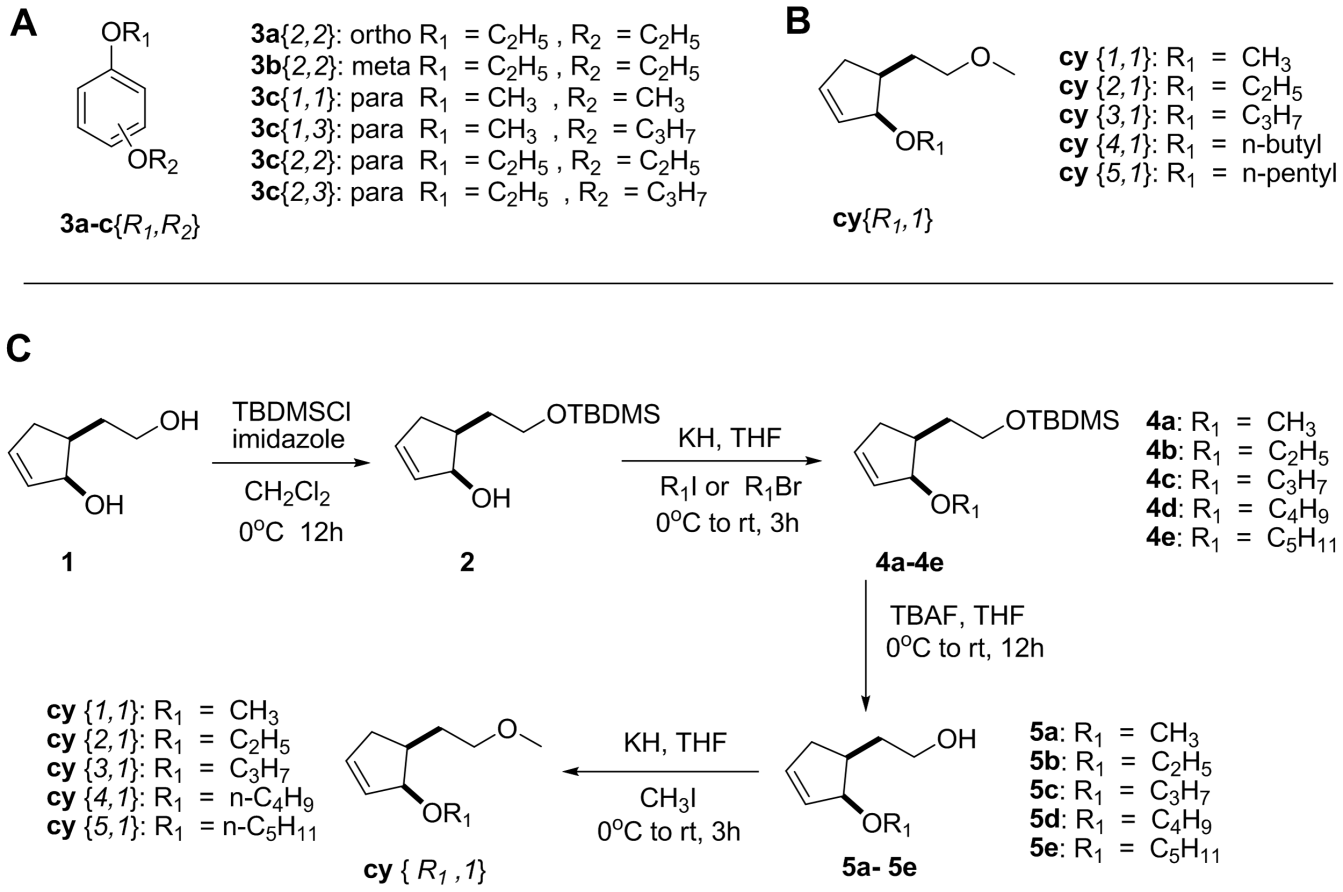
Compounds used in this study. **A**. Structures of the dialkoxybenzenes; their codenames are explained in ref [17]. **B**. Structures of the 5(2′-methoxyethyl) cyclopent-2-en-1-alkoxy diethers (**cy**{*R_1_,1*} compounds). **C**. Synthesis of the **cy**{*R_1_,1*} compounds. Abbreviations: rt =  room temperature; TBDMSCl  =  *tert*-butyl dimethylsilyl chloride; THF  =  tetrahydrofuran.

The compounds tested included six dialkoxybenzenes (one *ortho*, one *meta* and four *para* substituted) (Fig.1A), a 5-compound library of dialkyl ethers of *cis* 5-(2′-hydroxyethyl) cyclopent-2-en-1-ol (**cy**{*1-5,1*} code HC 2–169) and the individual library members (Fig.1B). The dialkoxybenzenes were synthesized as described in Paduraru et al. [17], whereas the alicyclic ethers, **cy**{*R_1_,R_2_*}, were synthesized as described in Chen et al. [18] and in Chen and Plettner [27]. Library HC 2–169 was “Library C1” from Chen et al [18]. Briefly (Fig. 1C), diol **1** was prepared as described in Chen et al. [28]. The diol **1** was singly protected by reaction with tert-butyldimethylsilyl chloride (TBDMSCl) and imidazole in dichloromethane [10], [27]. The monoprotected diol **2** was reacted with potassium metal in tetrahydrofuran (THF), followed by addition of the appropriate alkyl bromide or iodide (R_1_Br or R_1_I), resulting in compound **3**. This intermediate was deprotected using tetrabutylammonium fluoride (TBAF) in THF, to give compound **4**. This compound was reacted with potassium in THF, followed by iodomethane, to give the final product **cy**{*R_1_,1*}.

### Synthetic procedures and spectroscopic data of the racemic cy{*R_1_, 1*} compounds

#### Synthesis of (±)–*cis*-5-[2′-(*tert*-butyl dimethylsilanyloxy)-ethyl] cyclo-pent-2-enol (2)

A solution of compound **1** (1.5 g, 11.7 mmol), triethylamine (1.42 g, 14.0 mmol), N,N-dimethylaminopyridine, DMAP (142 mg, 1.17 mmol), *tert*-butyldimethylsilyl chloride (2.11 g, 14.0 mmol) in CH_2_Cl_2_ was stirred at 0°C for 12 h. The reaction mixture was diluted with CH_2_Cl_2_ and washed with water and brine. The organic layer was dried over MgSO_4_ filtered and concentrated under vacuum. The crude product was purified by flash chromatography on silica gel (EtOAc/ hexane 3: 7) to afford pure alcohol **2** as a colorless oil (2.26 g, 80%).

^1^H NMR (400 MHz, CDCl_3_) δ_H_ 6.05 (m,1 H), 5.95 (m,1H), 4.72 (dt, J = 7.2, 1.8 Hz, 1H), 3.71 (m, 1H), 3.55 (m, 1H), 3.07 (d, J = 3.2,-OH), 2.49–2.41(dddd, J = 12.0, 9.6, 4.8, 2.9 Hz, 1H), 2.29–2.21 (ddd, J = 14.5, 13.0, 13.0 Hz, 1H), 2.20–2.12 (dddd, J = 11.4, 9.4, 4.4, 2.8 Hz, 1H), 2.01–1.92 (m, 1H), 1.84–1.73 (ddd, J = 14.4, 12.9, 12.9 Hz, 1H), 0.89 (s, 9H), 0.02 (s, 3H), 0.00 (s, 3H).

#### General procedure for the preparation of compounds 4b–4e

A solution of compound **2** (1 mmol) in 6 ml of dry THF was added dropwise to a suspended solution of KH (1.1 mmol) in 20 ml of dry THF at 0°C. The mixture was stirred at 0°C for 30 min. The alkylating reagents (2.2 mmol) were added dropwise at 0°C. Once addition of the alkylating agent was completed, the reaction mixture was warmed to room temperature and kept stirring for another 3 h. The reaction was quenched with saturated NH_4_Cl solution. The organic solution was dried over MgSO_4_ and concentrated *in vacuo*. The residue was purified by flash chromatography on silica gel (hexanes/ EtOAc 9∶1) to give the desired compound.

#### Synthesis of Compound 4b

Compund **2** (500 mg, 2.07 mmol) was treated with KH (90 mg, 2.272 mmol) and bromoethane (450 mg, 4.132 mmol), according to the general method described above, to give pure product **4b** (colourless oil, 400 mg, 72%). ^1^H NMR(CDCl_3,_ 400 MHz) δ_H_ 6.04 (m, 1H), 5.94 (m, 1H), 4.25 (dt, J = 7.2, 1.8Hz, 1H), 3.71 (m, 2H), 3.44 (m, 1H), 3.32 (m, 1H), 2.49–2.41(dddd, J = 12.0, 9.6, 4.8, 2.9 Hz, 1H), 2.29–2.21 (ddd, J = 14.5, 13.0, 13.0 Hz, 1H), 2.20–2.12 (dddd, J = 11.4, 9.4, 4.4, 2.8 Hz, 1H), 2.01–1.92 (m, 1H), 1.84–1.73 (ddd, J = 14.4, 12.9, 12.9 Hz, 1H), 1.17 (t, J = 7.2 Hz, 3H), 0.89 (s, 9H), 0.02 (s, 6H).

#### Synthesis of Compound 4c

Compund **2** (500 mg, 2.07 mmol) was treated with KH (90 mg, 2.27 mmol) and 1-bromopropane (507 mg, 4.128 mmol), according to the general method described above, to give pure product **4c** (colourless oil, 400 mg, 68%). ^1^H NMR(CDCl_3,_ 400 MHz)δ_H_ 6.01 (m, 1H), 5.95 (m, 1H), 4.23 (dt, J = 7.2, 1.8 Hz, 1H), 3.69 (m, 2H), 3.44 (m, 1H), 3.32 (m, 1H), 2.49–2.41(dddd, J = 12.0, 9.6, 4.8, 2.9 Hz, 1H), 2.29–2.21 (ddd, J = 14.5, 13.0, 13.0 Hz, 1H), 2.20–2.12 (dddd, J = 11.4, 9.4, 4.4, 2.8 Hz, 1H), 2.01–1.92 (m, 1H), 1.84–1.73 (ddd, J = 14.4, 12.9, 12.9 Hz, 1H), 1.53–1.63 (m, 2H), 0.91 (t, J = 7.2 Hz, 3H), 0.90 (s, 9H), 0.02 (s, 6H).

#### Synthesis of Compound 4d

Compund **2** (500 mg, 2.07 mmol) was treated with KH (90 mg, 2.27 mmol) and 1-bromobutane (566 mg, 4.132 mmol), according to the general method described above, to give pure product **4d** (colourless oil, 425 mg, 69%). ^1^H NMR(CDCl_3,_ 400 MHz) δ_H_ 6.01 (m, 1H), 5.95 (m, 1H), 4.22 (dt, J = 7.2, 1.8 Hz, 1H), 3.68 (m, 2H), 3.44 (m, 1H), 3.32 (m, 1H), 2.43–2.35 (dddd, J = 12.0, 9.6, 4.8, 2.9 Hz, 1H), 2.34–2.25 (ddd, J = 14.5, 13.0, 13.0 Hz, 1H), 2.20–2.13 (dddd, J = 11.4, 9.4, 4.4, 2.8 Hz, 1H), 2.01–1.92 (m, 1H), 1.73–1.64 (ddd, J = 14.4, 12.9, 12.9 Hz, 1H), 1.61–1.51 (m, 2H), 1.43–1.33 (m, 2H), 0.91 (t, J = 7.2 Hz, 3H), 0.90 (s, 9H), 0.01 (s, 6H).

#### Synthesis of Compound 4e

Compund **2** (500 mg, 2.07 mmol) was treated with KH (90 mg, 2.27 mmol) and 1-iodopentane (818 mg, 4.132 mmol), according to the general method described in above section, to give pure product **4e** (colourless oil, 439 mg, 68%). ^1^H NMR(CDCl_3,_ 400 MHz) δ_H_ 6.01 (m, 1H), 5.95 (m, 1H), 4.22 (dt, J = 7.2, 1.8 Hz, 1H), 3.69 (m, 2H), 3.45 (m, 1H), 3.32 (m, 1H), 2.43–2.35 (dddd, J = 12.0, 9.6, 4.8, 2.9 Hz, 1H), 2.34–2.25 (ddd, J = 14.5, 13.0, 13.0 Hz, 1H), 2.20–2.13 (dddd, J = 11.4, 9.4, 4.4, 2.8 Hz, 1H), 2.01–1.92 (m, 1H), 1.73–1.64 (ddd, J = 14.4, 12.9, 12.9 Hz, 1H), 1.60–1.52 (m, 2H) 1.37–1.28 (m, 4H), 0.91 (t, J = 7.2 Hz, 3H), 0.90 (s, 9H), 0.01 (s, 6H).

#### General procedure for synthesis of compounds 5b–5e

To a stirred solution of compounds **4b**–**4e** (1–2 mmol) in 10 ml of THF was added tert-butyl ammonium fluoride (TBAF) (2 mmol) at room temperature. After 12 h, the reaction mixture was diluted with EtOAc, the organic layer was separated and washed with saturated NH_4_Cl and brine solutions. The organic layer was dried over MgSO_4_ and concentrated *in vacuo*. The crude product was purified by flash column chromatography on silica gel (hexanes/ EtOAc 7: 3) to afford the desired product.

#### General procedure for the racemic cy{*R_1,_ 1*} compounds

A solution of compounds **4b**–**4e** (1 mmol) in 6 ml of dry THF was added dropwise to a suspended solution of KH (1.1 mmol) in 20 ml of dry THF at 0°C. The mixture was stirred at 0°C for 30 mins. The alkylating reagents (2.2 mmol) were added dropwise at 0°C. Once addition of the alkylating agent was complete, the reaction mixture was warmed to room temperature and kept stirring for another 3 h. The reaction was quenched with saturated NH_4_Cl solution. The organic solution was dried over MgSO_4_ and concentrated *in vacuo*. The residue was purified by flash chromatography on silica gel (hexanes/ EtOAc 9∶1) to give the desired compound.

#### Synthesis of cy{*1,1*}

Compound **1** (100 mg, 0.78 mmol) was treated with KH (34 mg, 0.859 mmol) and iodomethane (424 mg, 3.124 mmol), according to the general method described in the section above, to give pure product **cy**{*1,1*} (colourless oil, 30 mg, 25%). ^1^H NMR(CDCl_3,_ 400 MHz) δ_H_ 6.01 (m, 1H), 5.95 (m, 1H), 4.14 (dt, J = 7.2, 1.8 Hz, 1H), 3.48 (td, J = 6.4, 2.1 Hz, 1H), 3.37 (s, 3H), 3.34 (s, 3H), 2.43–2.35 (dddd, J = 12.0, 9.6, 4.8, 2.9 Hz, 1H), 2.34–2.25 (ddd, J = 14.5, 13.0, 13.0 Hz, 1H), 2.20–2.13 (dddd, J = 11.4, 9.4, 4.4, 2.8 Hz, 1H), 2.01–1.99 (m, 1H), 1.73–1.64 (ddd, J = 14.4, 12.9, 12.9 Hz, 1H); MS m/z (relative intensity): 157 (M+1,10%), 149 (25%), 109 (50%), 69 (100%).

#### Synthesis of cy{*2, 1*}

Compound **5b** (250 mg, 1.6 mmol) was treated with KH (76 mg, 19.2 mmol) and iodomethane (454 mg, 3.2 mmol), according to the general method described above, to give pure product **cy**{*2, 1*} (colourless oil,220 mg, 81%). ^1^H NMR(CDCl_3,_ 400 MHz) δ_H_ 6.01 (m, 1H), 5.94 (m, 1H), 4.25 (dt, J = 7.2, 1.8 Hz, 1H), 3.60–3.52 (m, 1H), 3.52–3.43 (m, 3H), 3.37 (s, 3H), 2.43–2.35 (dddd, J = 12.0, 9.6, 4.8, 2.9 Hz, 1H), 2.34–2.25 (ddd, J = 14.5, 13.0, 13.0 Hz, 1H), 2.20–2.13 (dddd, J = 11.4, 9.4, 4.4, 2.8 Hz, 1H), 2.01–1.92 (m, 1H), 1.73–1.64 (ddd, J = 14.4, 12.9, 12.9 Hz, 1H), 1.20 (t, J = 7.2 Hz, 3H); MS m/z (relative intensity): 171 (M+1, 1%), 169 (M-1, 25%), 125 (100%).

#### Synthesis of cy{*3, 1*}

Compound **5c** (440 mg, 2.58 mmol) was treated with KH (120 mg, 3 mmol) and iodomethane (734 mg, 5.176 mmol), according to the general method described above, to give pure product **cy**{*3, 1*} (colorless oil, 300 mg, 63%). ^1^H NMR(CDCl_3,_ 400 MHz) δ_H_ 6.01 (m, 1H), 5.95 (m, 1H), 4.23 (dt, J = 7.2, 1.8Hz, 1H), 3.55–3.42 (m, 3H), 3.41–3.33 (m, 1H), 3.38 (s, 3H), 2.43–2.35 (dddd, J = 12.0, 9.6, 4.8, 2.9 Hz, 1H), 2.34–2.25 (ddd, J = 14.5, 13.0, 13.0 Hz, 1H), 2.20–2.13 (dddd, J = 11.4, 9.4, 4.4, 2.8 Hz, 1H), 2.01–1.92 (m, 1H), 1.73–1.64 (ddd, J = 14.4, 12.9, 12.9 Hz, 1H), 1.63–1.54 (m, 2H), 0.91 (t, J = 7.2 Hz, 3H); MS m/z (relative intensity): 185 (M+1, 1%), 125 (75%), 93 (100%).

#### Synthesis of cy{*4, 1*}

Compound **5d** (500 mg, 2.71 mmol) was treated with KH (130 mg, 3.26 mmol) and iodomethane (771 mg, 5.43 mmol), according to the general method described above, to give pure product **cy**{*4, 1*} (colorless oil, 438 mg, 82%). ^1^H NMR (CDCl_3,_ 400 MHz) δ_H_ 6.01 (m, 1H), 5.95 (m, 1H), 4.22 (dt, J = 7.2, 1.8 Hz, 1H), 3.54–3.44 (m, 3H), 3.41 (m, 1H), 3.38 (s, 3H), 2.43–2.35 (dddd, J = 12.0, 9.6, 4.8, 2.9 Hz, 1H), 2.34–2.25 (ddd, J = 14.5, 13.0, 13.0 Hz, 1H), 2.20–2.13 (dddd, J = 11.4, 9.4, 4.4, 2.8 Hz, 1H), 2.01–1.92 (m, 1H), 1.73–1.64 (ddd, J = 14.4, 12.9, 12.9 Hz, 1H), 1.61–1.51 (m, 2H), 1.43–1.33 (m, 2H), 0.91 (t, J = 7.2 Hz, 3H); MS m/z (relative intensity): 197 (M-1, 2.5%), 125 (25%), 109 (75%), 93 (100%).

#### Synthesis of cy{*5, 1*}

Compound **5e** (280 mg, 1.414 mmol) was treated with KH (67 mg, 1.696 mmol) and iodomethane (401 mg, 2.228 mmol), according to the general method described above, to give pure product **cy**{*5,1*} (colorless oil, 220 mg, 74%). ^1^H NMR (CDCl_3,_ 400 MHz) δ_H_ 6.01 (m, 1H), 5.95 (m, 1H), 4.22 (dt, J = 7.2, 1.8 Hz, 1H), 3.53–3.44 (m, 3H), 3.43–3.36 (m, 1H), 3.38 (s, 3H), 2.43–2.35 (dddd, J = 12.0, 9.6, 4.8, 2.9 Hz, 1H), 2.34–2.25 (ddd, J = 14.5, 13.0, 13.0 Hz, 1H), 2.20–2.13 (dddd, J = 11.4, 9.4, 4.4, 2.8 Hz, 1H), 2.01–1.92 (m, 1H), 1.73–1.64 (ddd, J = 14.4, 12.9, 12.9 Hz, 1H), 1.60–1.52 (m, 2H) 1.37–1.28 (m, 4H), 0.91 (t, J = 7.2 Hz, 3H); MS m/z (relative intensity): 211 (M-1, 2.5%), 159 (50%), 91(100%).

### Electrophysiology bioassay

Electrophysiological (EP) recordings were carried out on the olfactory sensory organ on the *Varroa* foreleg. The foreleg was dissected at the base and mounted between two glass capillaries filled with KCl solution (0.1 N), each containing a silver recording electrode thus closing the electrical circuit. A constant flow of charcoal-filtered and humidified air was blown towards the organ at a rate of 100 ml/min using a stimulus flow controller (model CS-05; Syntech, Hilversum, the Netherlands).

**Figure 2:**
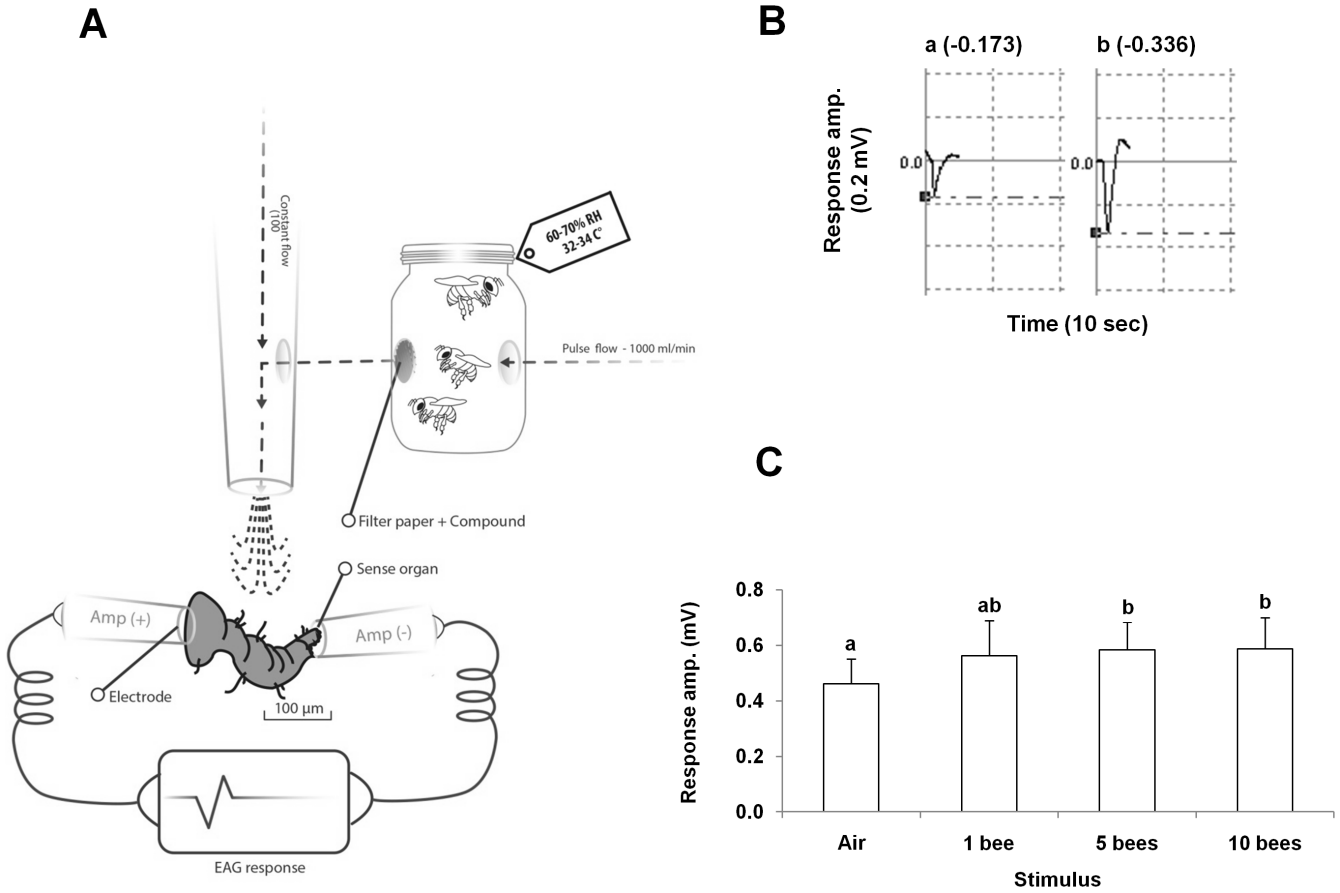
Electrophysiology with the *Varroa* foreleg. **A**. Electrophysiology setup of an isolated *Varroa* foreleg that was stimulated with the headspace volatiles of freshly caught honey bees in a jar. **B**. Typical traces of *Varroa* foreleg responses to air (left) and honey bee volatiles (right). **C**. *Varroa* foreleg electrophysiological response amplitude. Comparison between the responses to the headspaces of different numbers of bees: no bee (empty jar), 1, 5 and 10 bees. ANOVA repeated measures: bars marked by different letters are significantly different, p<0.05, n = 6.

The effect of the disruptive compounds on the EP response was measured relative to the response to a positive stimulus (honey bee headspace). The headspace was presented by puffing charcoal-filtered air (1000 ml/min, for 1 second) through a glass jar that contained freeze-killed nurse bees (1, 5 or 10 bees were tested) kept in a controlled environment (32–34C°, 62–70%). Head space of an empty jar kept under the same conditions was used as control (Figure 2). Each foreleg was used to test all the treatments.

To prepare EP cartridges of the potential disrupting compounds, 1 µl of the compound dissolved in hexane was pipetted onto a piece of filter paper (Whatman No 1) which is placed in a glass Pasteur pipette and exposed to air for 30 s to allow solvent to evaporate. Three different stimuli were tested: a “positive stimulus” (five bees' headspace), a “positive stimulus+compound”, a control stimulus- “Air” (an empty jar) and hexane.

**Figure 3:**
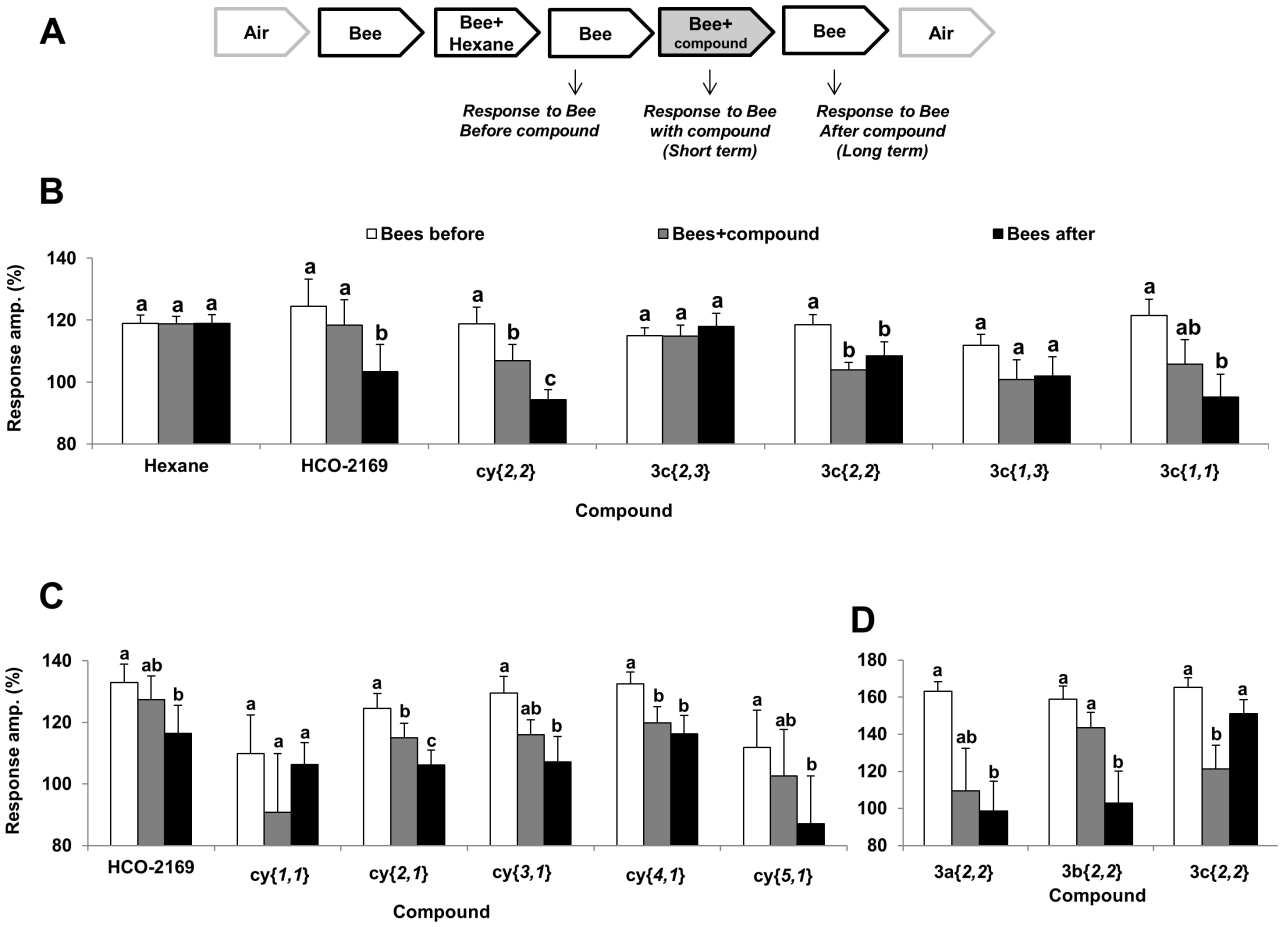
Electrophysiological screening of the compounds. **A**. Order of the *Varroa* foreleg stimulations and terminology used for the corresponding responses. The time interval between each stimulus was 30 s, unless otherwise stated. The stimuli were: Air, Headspace of five nurse bees (bee stimulus), Bee stimulus together with the compound (Bee stimulus + comp) or of the hexane control (Bee stimulus + hexane). In italics, below the stimuli, are the names of the values presented in the results. **B**. Initial screen of the *Varroa* foreleg electrophysiological response to different stimuli, all loaded at 10 µg in the stimulus cartridge (normalized values against the response to air %, average+SE). For the bee stimuli, the headspace from 5 nurse bees was used. Bars marked by different letters are significantly different, ANOVA repeated measures, p<0.05, n = 10.**C**. Testing of the individual components of the blend HCO-2169 at 10 µg doses (n = 10). **D**. Experiment with the three isomers of diethyoxybenzene at 10 µg doses (n = 6).

In all experiments, the stimuli were given in the same order on the same forelegs as presented in Fig. 3A. When more than one compound was tested, the experiments with the different compounds were done in a random order. At least six different *Varroa* forelegs were tested (one from each individual) for each experiment.

The EP response (mV) was amplified and recorded by a PC via an IDAC-232 for data acquisition using the “EAG 2000” and “GCEAD-2000” softwares (all Syntech). For the organ to recover and to prevent adaptation, we allowed intervals of 30 s between each stimulus unless specified otherwise.

The response amplitude was normalized relative to the response of the same organ to the control stimulus (eq. 1). Only individuals that showed a higher response to the “positive stimulus” than to the control stimulus prior to the exposure to the compounds were used for statistical analysis. The effect of the compounds was evaluated relative to the response to the “positive stimulus” before the exposure to the tested compound. According to previous studies [19], [20], two kinds of effect were evaluated: the effect that occurred in the presence of the compound termed “short term effect”, and the effect following the administration of the compound but not in its presence termed “long term effect”.
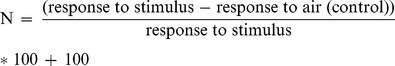



#### Normalization Equation 1

*N*- Response amplitude normalized relative to the response to air (%).

### Behavioral bioassays

The effect of the compounds on *Varroa* host preference was tested in a two-choice bioassay based on Kraus [10]. In the bioassay, a single mite was placed in the center of the arena (90 mm diameter and 17 mm deep glass Petri dish) and was presented with a choice of a freshly killed forager and a nurse (killed by freezing for one hour) placed on opposite sides of the arena. The experiments were conducted in a controlled dark environment, at 34–35°C and 60–70% RH (simulating conditions in a bee hive). The *Varroa* choice was examined in the presence of 0.01 µg, 0.1 µg and 10 µg of the compound dissolved in 1 µl hexane or in the presence of 1 µl pure hexane, as control. The compound or hexane, were placed right above the *Varroa* on the inner side of the cover plate, on a piece of parafilm for slow release (5*5 mm, Bemis, USA). Each dose was tested at least in 2 replicates; in each replicate 10 to 19 mites were tested for each treatment (Compound or Hexane). The mite position on a nurse, a forager or elsewhere was documented after 180 minutes. *Varroa* host preference between a forager and a nurse bee was calculated as the percentage of total mites reaching each of the hosts. *Varroa* ability to reach any of the hosts was calculated as the percentage of viable mites by the end of the experiment out of the total tested mites.

### Statistical analysis

For the electrophysiology assays, the original data in mV, or the normalized data in percentages were analyzed using ANOVA repeated measures, followed by a post hoc Tukey-Kramer test. A Bonferroni correction was used when needed.

For behavioral assays, logistic regression analysis was used to assess the dose effect of the compounds on Varroa host preference. The model included replicate effect (interaction between dose and replicate, was removed from the model due to non-significance). Odds ratio, 95% confidence intervals and p-values are reported. A possible effect of the compounds on *Varroa* ability to reach any of the hosts was assessed using Chi-square test on proportion of mites reaching any of the hosts, out of the viable mites. All Statistical procedures were carried out with the SAS JMP Start statistic program 7.0.2.

### Molecular modeling

Structures were drawn in ChemDraw and imported into ChemBio3D Ultra v. 11 (CambridgeSoft, Cambridge, Massachusetts, USA). Each model was first minimized with MM2, and then with PM3, a semi empirical method. In both minimizations all atoms were allowed to move freely, and PM3 minimizations were done with the closed shell wave function, the EF optimizer, until the RMS gradient was ≤0.1. Initial minimization was done in a vacuum, followed by minimization in water and in chloroform. No significant differences were seen between the three environments, so the structures obtained in chloroform (which mimics the hydrophobic environment of potential binding sites on or in proteins) were used for further exploration. To establish the breadth of the minima, double dihedral angle plots were constructed for all sets of neighboring C and/or O atoms around which free rotation is possible. Between dihedral angle explorations, the model was “heated” by short molecular dynamics trajectory (1000 iterations) at 700 K, after which the last structure was minimized again in PM3. For overlaying of structures, the global energy minima of the two structures were used, with the software's overlay algorithm. Overlays were done with **cy**{*4,1*} as the target structure or with **3b**{*2,2*} as the target. Both overlay simulations delineated a similar space.

Correlations between compound structure and activity were obtained using Molecular Operating Environment (MOE, Chemical Computing Group, Montréal, Canada). All compounds tested here and DEET were drawn and minimized using the “builder” function in MOE. The activities used were the difference between the “Bee before” and the “Bee + compound” treatments, for the Δ STI (%), or the difference between the “Bee before” and the “Bee after” treatments, for the Δ LTI (%). A molecular database was assembled in MOE with the compounds and their activities. Using the quantitative structure activity (QSAR) protocol and 19 parameters a structure activity model was calculated. The parameters calculated were: 1) dipole (AM1), 2) highest occupied molecular orbital HOMO (eV) (AM1), 3) lowest unoccupied molecular orbital LUMO (eV) (AM1), 4) sum of atomic polarizabilities, 5) total accessible surface area 6) positive accessible surface area, 7) negative accessible surface area, 8) total hydrophobic accessible surface area, 9) total polar accessible surface area, 10) fraction of rotable bonds, 11) density (atomic mass units/Å^3^) 12) angle bend energy, 13) van der Waals energy, 14) molecular globularity, 15) log of octanol/water partition coefficient (logP_o/w_), 16) mutagenicity (this algorithm scans the compounds for mutagenic groups), 17) heat of formation (kcal/mol)(PM3), 18) HOMO (eV) (PM3), 19) LUMO (eV) (PM3). All activity-parameter plots were checked for linear correlation in the entire set and, if relevant, within a subset of the compounds. Compound **cy**{*2,2*} was modeled as the opposite enantiomer than compounds **cy**{*R_1_,1*} (see below).

## Results

### Electrophysiology

To test the disruption of the *Varroa* host detection we selected nurses' headspace as a positive stimulus. Stimulation of the *Varroa* foreleg with headspace from different numbers of bees (1, 5 or 10) indicated that, although one honey bee head space elicited some response in the *Varroa* leg, only stimuli of 5 and 10 bees evoked significantly higher response than air (F(3, 15)  = 4.75, p = 0.016, ANOVA repeated measures followed by a Bonferroni correction; Fig. 2C). As 10 bees' headspace did not add a significant increase in the response amplitude we used the headspace of five bees in further experiments.

### *Varroa* responses to sequential stimuli of bee headspace

In order to check for a possible habituation of the *Varroa* foreleg to honey bee volatiles, and the response stability over time, sequential stimuli of five-bee headspace were puffed at intervals of 30 seconds. Comparing the response amplitudes in 7 different *Varroa* mites, no significant difference was found between the response amplitudes (F(2, 12)  = 0.0407, p = 0.96, ANOVA repeated measures; Fig. S1), and the response remained stable for at least 20 min.

### The Effect Of “Disrupting” Compounds On The *Varroa* Response To Bee Headspace

The disruptive effect of 6 different compounds on the electrophysiological response of *Varroa* foreleg to honey bee headspace was tested, by sequentially stimulating the foreleg with air, bee headspace or mixed bee headspace + compound stimuli (Fig. 3A). A significant inhibitory effect on the sensory organ was apparent for most of the tested compounds except for the hexane-control and **3c**{*2,3*} at 10 µg (ANOVA repeated measures, *p*<0.05). The impact of the inhibiting compounds on mite responses to honey bee headspace was not the same. A significant short-term inhibitory effect was found for compounds **3c**{*2,2*} and **cy**{*2,2*} (F(2, 16)  = 8.92, p = 0.002; F(2, 16)  = 42.8, p<0.0001), while a significant long-term effect was observed with **3c**{*1,1*}, **cy**{*2,2*} and the blend, HCO-2169 (F(2, 16)  = 3.89, p = 0.04; F(2, 16) = 19, p<0.0001) (Fig. 3B). This long-term inhibition appeared stronger than the short-term effect for **cy**{*2,2*} and HCO-2-169, but it was eliminated in a fourth stimulation with bee headspace that was applied after stimulation with air (data not shown). A similar result has been obtained with gypsy moths treated with the sex pheromone, (+)-disparlure, and a blend **3c**{*1-5,3*} [19]: the long-term inhibition was reversible, and the response to pure pheromone returned to normal 4–5 puffs after the mixed puff (pheromone +**3c**{*1-5,3*}). In the present study, there were different structure-activity relationships for the short-term and long-term effects. For example, **3c**{*1,1*}, **3c**{*2,2*} and **cy**{*2,2*} were similar in their short-term effect, whereas **3c**{*2,3*} was not active. In terms of the long-term effect the activity was: **cy**{*2,2*} >**3c**{*1,1*} ≅**3c**{*2,2*}; **3c**{*2,3*} was not active. Similarly, in Plettner's and Gries' study [19], the structure-activity relationships differed for short-term and long-term effects.

HCO-2-169 is a blend of methyl-substituted cy compounds: **cy**{*1-5,1*}. To reveal structure-activity relationship of the inhibitory effect, we focused on components of HCO-2-169: **cy**{*1,1*}, **cy**{*2,1*},**cy**{*3,1*}, **cy**{*4,1*} and **cy**{*5,1*}. The different components as well as the whole mixture (HCO-2-169, as a positive control) were tested in a random order. Except for **cy**{*1,1*}, all of the tested compounds had a long-term inhibiting effect on the *Varroa* response to bee headspace (Fig. 3C). The three most effective compounds in that series were: **cy**{*4,1*}, **cy**{*3,1*}and **cy**{*2,1*} (F(2, 18)  = 10.7, p = 0.0009; F(2, 18)  = 4.1, p = 0.03; F(2, 18)  = 14.6, p = 0.0002). To follow up on the structure-activity relationship of the dialkoxybenzenes, experiments with the three isomers of diethoxybenzene, **3a**{*2,2*}, **3b**{*2,2*} and **3c**{*2,2*}, as well as **cy**{*2,2*} and **3c**{*1,1*} were performed. The isomers of diethoxybenzene differed in their activity: **3c**{*2,2*} was the best short-term inhibitor but showed no long-term inhibition, whereas **3b**{*2,2*} and **3a**{*2,2*} exhibited a long term inhibition (F(2, 10)  = 9.9, p = 0.004; F(2, 10)  = 16.8, p = 0.001; F(2, 10)  = 5.9, p = 0.026) (Fig. 3D). Compound **cy**{*2,2*} showed moderate long-term inhibition (see below). For **3c**{*1,1*} the long-term inhibition at the higher doses of 1 or 10 µg was confirmed (Fig. S3).

**Figure 4:**
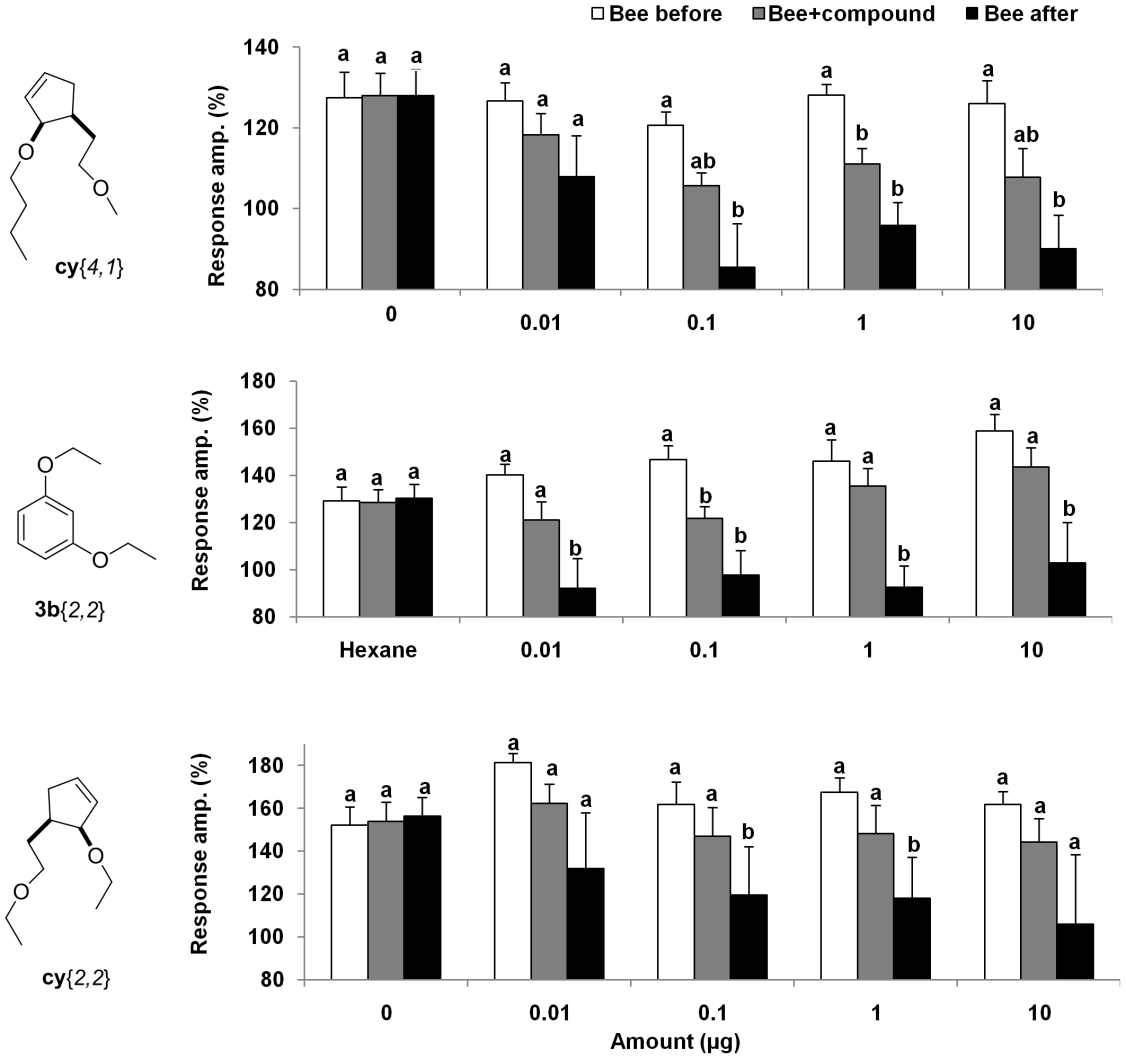
Dose responses of long-term inhibitory compounds cy{*4,1*}, 3b{*2,2*} and cy{*2,2*}. The responses of the *Varroa* forelegs to stimulation with different amounts of each compound and with the headspace from 5 nurse bees (normalized values against the response to air %, average+SE). Bars within each dose, marked by different letters, are significantly different, ANOVA repeated measures, p<0.05, n = 7.

**Figure 5:**
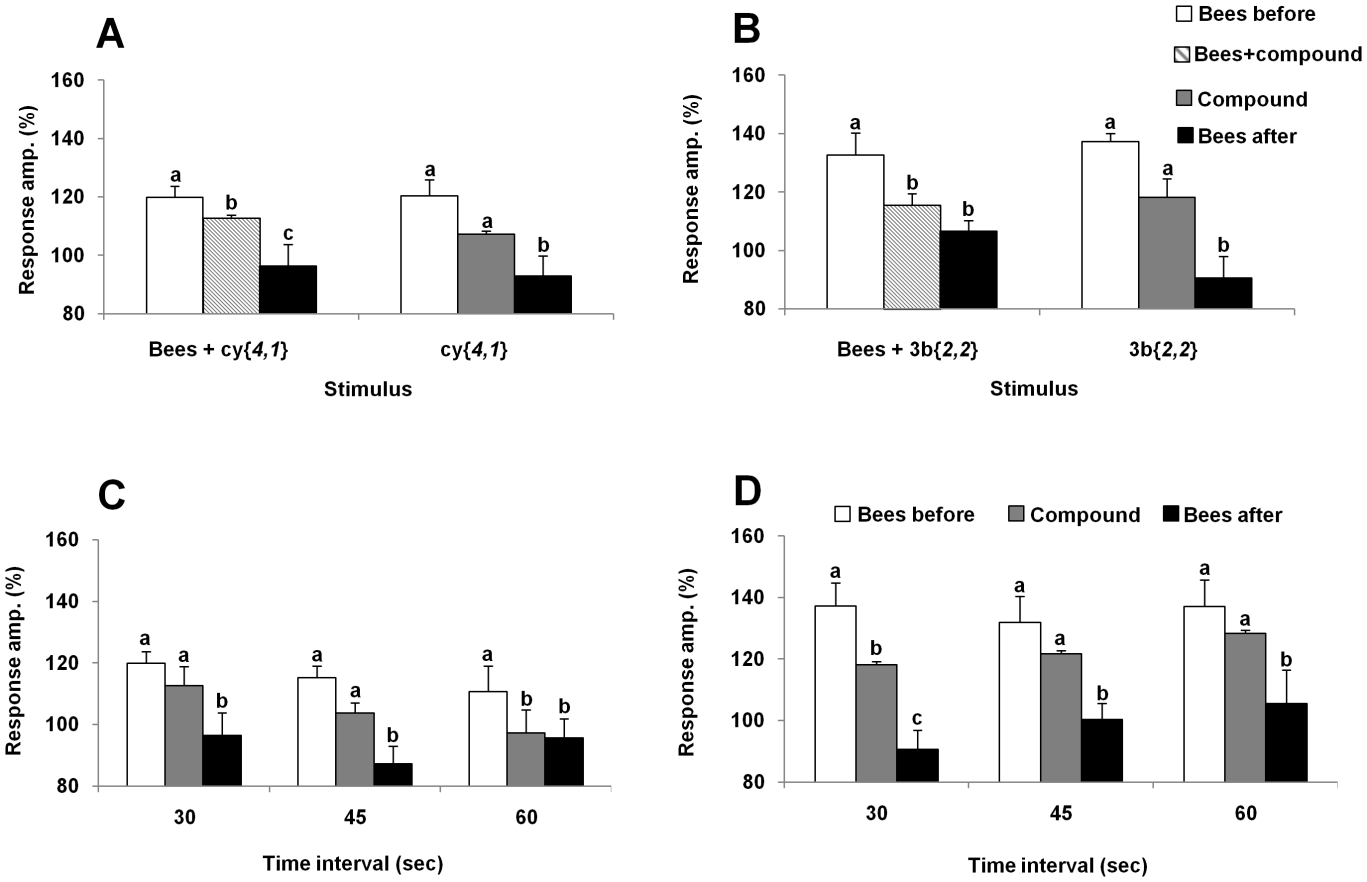
Detailed evaluation of the long-term inhibitory effect of the most active compounds. The effect of 0.1 µg **cy**{*4,1*} (**A**) or **3b**{*2,2*} (**B**), with and without a simultaneous stimulus of the headspace volatiles from 5 nurse bees, on the electrophysiological response of the *Varroa* foreleg. The data are normalized values (%, average+SE): bars marked by different letters are significantly different, ANOVA repeated measures, p<0.05, p<0.05, n = 6. The longevity of the inhibitory effect of 0.1 µg **cy**{*4,1*} (**C**) or 0.1 µg **3b**{*2,2*} (**D**) on *Varroa* foreleg electrophysiological responses. The time interval between the mixed stimulus (Bee + compound) and the pure bee stimulus was varied. Values are normalized against the response to air (%, average+SE): bars marked by different letters are significantly different, ANOVA repeated measures, p<0.05; n = 6.

We chose to further evaluate the specificity of the *Varroa* leg response to **cy**{*4,1*}, because it is less volatile than **cy**{*2,1*}, **cy**{*3,1*} or **3b**{*2,2*} and therefore easier to work with. First, a dose response was measured. The dose of 0.01 µg was inactive both short and long-term (F(2, 12)  = 2.9, p = 0.08). Doses of 0.1 µg and higher were all active long-term, and short-term only for the 1 µg dose (F(2, 12)  = 14.9, p = 0.0005) (Fig. 4). The optimal dose appears to be 0.1 µg. This dose was used in subsequent experiments with **cy**{*4,1*}. Similarly, dose responses were obtained for the other two long-term inhibitors, **cy**{*2,2*} and **3b**{*2,2*}. For **3b**{*2,2*} a long-term inhibition was found for all of the doses (0.01 µg F(2, 10)  = 20.4, p = 0.0001; 0.1 µg F(2, 10)  = 15.4, p = 0.001; 1 µg F(2, 10)  = 23.4, p = 0.0001; 10 µg F(2, 10)  = 16.8, p = 0.001), while a short-term inhibition was observed only for a dose of 0.1 µg. On the other hand, **cy**{*2,2*} was only long-term active at doses of 0.1 µg and 1 µg (F(2, 10)  = 13, p = 0.002; F(2, 10)  = 9.7, p = 0.005). When 0.1 µg of **cy**{*4,1*} stimulus was given alone, the compound elicited a response that was not significantly different from the honey bee head space (Fig. 5A). However, subsequent stimulation with honey bee headspace was significantly inhibited long-term (F(2,10)  = 14.3, p = 0.001), similarly to the situation when both stimuli were applied together (F(2,10)  = 25.6, p = 0.0001). A similar effect was found with the stimulus of **3b**{*2,2*} alone (F(2,14) = 18.4, p<0.0001), and when given in the presence of the bee headspace (F(2,14) = 9.06, p = 0.003). This activity differs from the effect of long-term inhibitors studied with gypsy moth antennae, in that those compounds were only inhibitory after a mixed stimulus and not by themselves [19]. We examined the longevity of the inhibitory effect of **cy**{*4,1*} and **3b**{*2,2*} by varying the time interval between the two sets of stimuli: “compound” and “bees after compound”: 30, 45 or 60 s. The results suggest that the effect of both compounds lasts for at least 60 s, (**cy**{*4,1*}: 30 s- F(2, 10)  = 14.3, p = 0.001; 45 s- F(2, 10)  = 19.4, p = 0.0004; 60 s -F(2, 10)  = 11.8, p = 0.002, and for **3b**{*2,2*} 30 s- F(2, 12)  = 26.5, p<0.0001; 45 s- F(2, 12)  = 23.6, p<0.0001; 60 s- F (2, 12)  = 13.5, p = 0.001) (Fig. 5B).

### The Effect Of Eag Inhibiting Compounds On *Varroa* Host Selection

**Figure 6:**
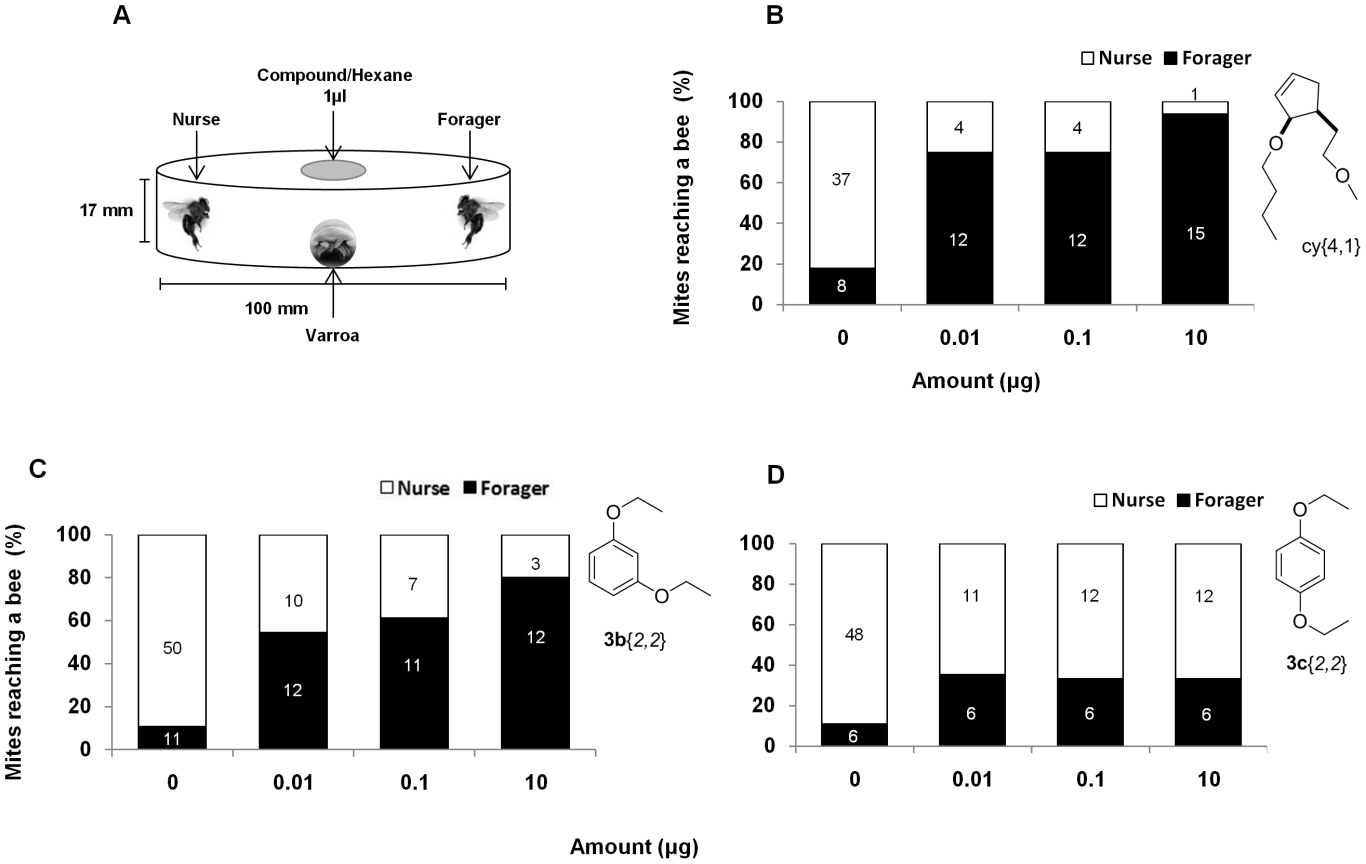
The effect of selected compounds on *Varroa* host choice between a nurse and a forager bee. **A**. Experimental setup. The test compound did not contact the mite, and the mite could move around and choose between a freshly killed nurse or forager. **B**. Effect of **cy**{*4,1*}: data are the percentage of *Varroa* that selected a particular host in the presence of hexane (control) or disrupting compound **cy**{*4,1*} at different doses (0.01 µg, 0.1 µg, 10 µg). Numbers within the bars show the number of *Varroa* choosing each of the hosts. **C**. Effect of **3b**{*2,2*}. **D**. Effect of **3c**{*2,2*}.

The mites' choice for nurse or a forager bee was significantly dependent on the treatment. As can be seen in Fig. 6A, after 180 min, in the presence of a solvent hexane (control) most of the mites (84%) chose the nurse bee, whereas in the presence of disrupting compound **cy**{*4,1*} only a minority of mites chose the nurse bee over the forager. The extent to which foragers were chosen over nurses was dose dependent: at 10 µg, about 94% of *Varroa* were found on the forager bee, while at 0.1 µg and 0.01 µg doses 75% and 71% of *Varroa* mites chose the forger bee, respectively (OR = 2.3, (95% CI 1.7–3.5), p<0.0001; Fig. 6B). Compounds **3b**{*2,2*} and **cy**{*2,2*} exhibit a similar activity (OR = 1.8, (95% CI 1.5–2.4), p<0.0001; OR = 2, (95% CI 1.6–2.5), p<0.0001; Fig. 6C and Fig. S2), whereas compound **3c**{*2,2*} did not alter the natural preference of the mites for nurse bees over foragers, yet it had a significant effect in reducing nurse preference (OR = 1.4, (95% CI 1.1–1.8), p = 0.002; Fig. 6D).

**Figure 7:**
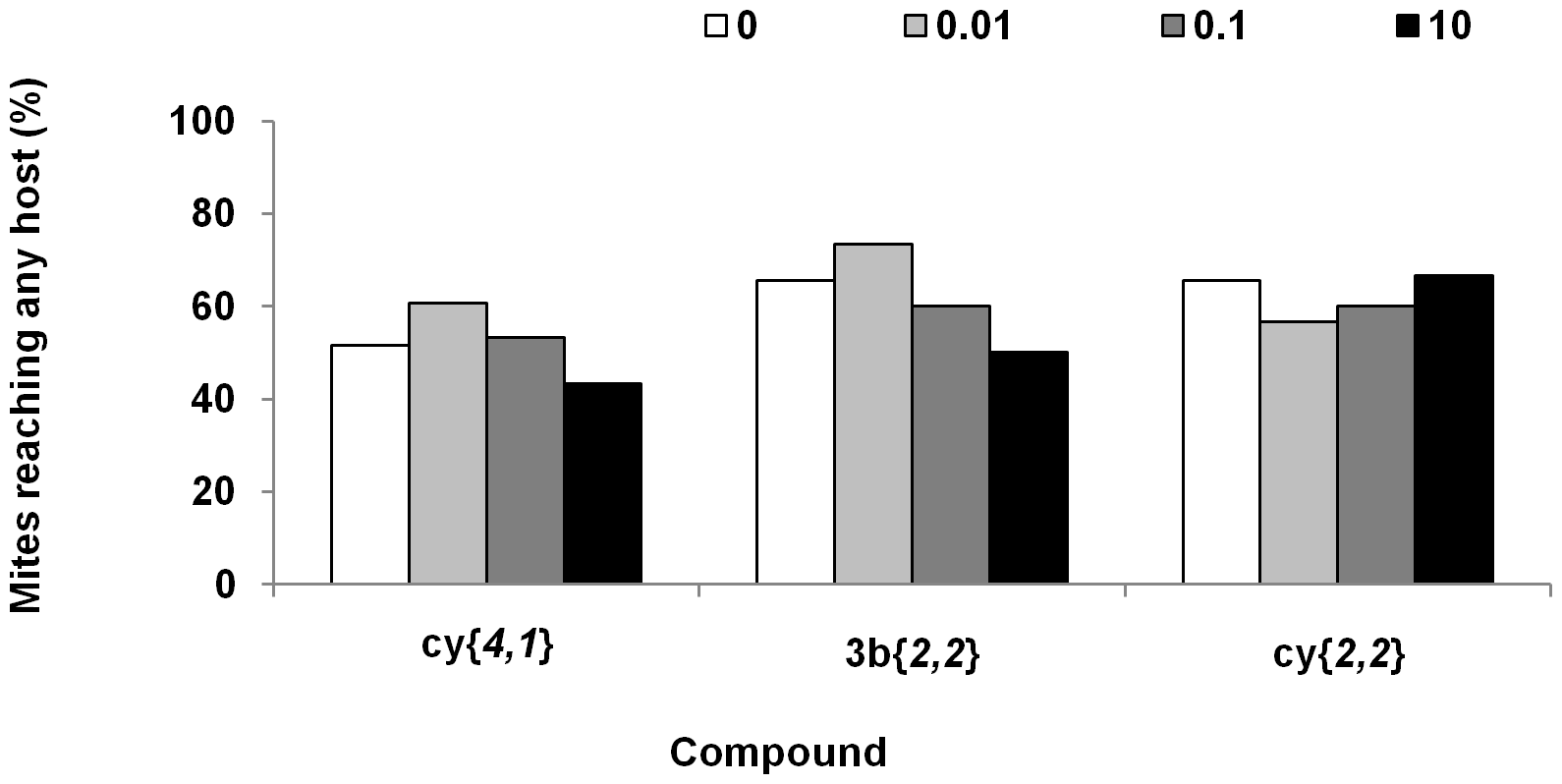
The effect of selected compounds on *Varroa* ability to reach any host. Effect of 3 selected compounds on the percentage of mites reaching any of the hosts in the choice bioassay, 180 min from the beginning of the experiment. The data are percentage of viable mites in the presence of hexane (control) or disrupting compound at each of three tested doses (0.01 µg, 0.1 µg, 10 µg) Chi-square test, ns.

*Varroa* starts dispersing shortly after the beginning of the experiment, but even after 180 minutes only 43–73% of mites reached any of the hosts (Fig. 7). Only a few died during the experiment. However, there was no significant reduction in the ability of mites to reach any of the hosts in any of the treatments (Chi-square test, **cy**{*4,1*} χ^2^ (3) = 2.01, n = 192, p = 0.57; **3b**{*2,2*} χ^2^ (3) = 3.9, n = 180, p = 0.27; **cy**{*2,2*} χ^2^ (3) = 1.04, n = 180, p = 0.79).

### Structure-Activity Relationship

**Figure 8:**
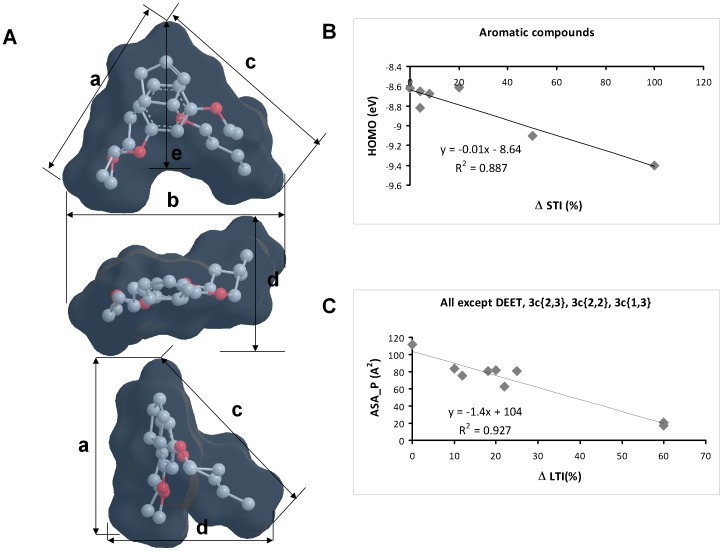
Active space and structure-activity of host choice alteration activity. **A**. Overlay of energy minimized conformers of **cy**{*4,1*} and **3b**{*2,2*}. The Connolly molecular surface of the overlaid molecules is shown in light blue. Hydrogen atoms and lone pairs have been omitted on the structures, but are included in the surfaces. Distances: a∼8.6 Å, b∼10 Å, c∼8.5 Å, d∼6.9 Å, e∼5.9 Å. **B** and **C**. Examples of the two structure-activity correlations found. **B**. Correlation between the highest occupied molecular orbital (HOMO) energy and the difference in short-term inhibition (%) between the “Bee before” and “Bee + compound” treatments (Δ STI (%)). Only the aromatic compounds (**3c** series, **3a**{*2,2*}, **3b**{*2,2*} and DEET) are included. **C**. Correlation between the polar accessible surface area (ASA_P) and the difference in long-term inhibition (%) between the “Bee before” and “Bee after” treatments (Δ LTI (%)).

Compounds **cy**{*4,1*} and **3b**{*2,2*} were the most active congeners for both, long-term inhibition and mite host selection alteration. Assuming that they exert their effects at or near their energy minima, a distorted “V-shaped” active space is delineated by the overlaid structures of **cy**{*4,1*} and **3b**{*2,2*} (Fig. 8A). The epitopes that seem to confer activity are: 1) a planar or nearly planar ring with π electron density, 2) the oxygen atoms of the ether moieties and 3) the alkyl substituents. The two most active compounds can place the ring and both oxygens in similar regions, relative to each other (Fig. 8A). Inactive compounds either do not fill the site (*e.g.* **cy**{*1,1*}) or cannot place both oxygens and the ring in the regions required for activity. *E.g.*, **3c**{*1,3*}, **3c**{*2,2*}, **3c**{*2,3*} were all inactive in both, long-term inhibition and mite host selection alteration. There appears to be some flexibility as to the extent to which both ether alkyl substituent pockets are filled. For example, **cy**{*2,1*} and **cy**{*3,1*} were both active as long-term inhibitors. However, there is a limit as to the size of the group the alkyl pockets can accommodate: **cy**{*5,1*} was slightly less active than **cy**{*4,1*} with regard to long-term inhibition, suggesting that a plateau had been reached (Figures 3 and 4). Compound **cy**{*2,2*} presents an interesting case: it can only be fit into the active space with the stereochemistry at both chiral centers reversed. Even then, the ethyl group at position 1 of the cyclopentene ring projects outside of the alkyl pocket and, more importantly, the oxygen atoms are located at different positions from those in the overlaid **cy**{*4,1*} and **3b**{*2,2*} space. Thus, compound **cy**{*2,2*} is slightly less active than **cy**{*4,1*} (Figures 4, 6B and S2), and the enantiomer that is active should be opposite to the active **cy**{*4,1*} enantiomer. The enantiomerically pure compounds will be tested in the future.

DEET (3-methyl-N,N-diethylbenzamide, a well-known insect repellent) has been tested in a separate study, and was found only to long-term inhibit the *Varroa* response to bee headspace at a high dose, while some short-term inhibition was also there, albeit not significant (Singh et al, submitted). Interestingly, DEET does not fit into the active space delineated by the two most active long-term inhibitors and host-preference-altering compounds, **cy**{*4,1*} and **3b**{*2,2*} (Fig. S4).

The molecular properties (parameters) in the structure activity model that correlated with activity differed between the short-term inhibition and long-term inhibition effects (Table S1). Only parameters shown in Table S1 correlated with activity; all others did not. The aromatic compounds (**3c**{*1,1*}, **3c**{*1,3*}, **3c**{*2,2*}, **3c**{*2,3*}, **3a**{*2,2*},**3b**{*2,2*} and DEET) showed weak linear correlation between the highest occupied molecular orbital (HOMO) and short-term inhibitory activity (Fig. 8B). This line had a negative slope, suggesting that the lower the energy of the HOMO, the more active. The energy of the LUMO was nearly constant (especially for the **3c** compounds), such that the larger the LUMO-HOMO gap, the more active the compound.

For the long-term inhibition, several relationships were found with electronic, conformational and surface properties (Table S1). All **3c** compounds showed a negative correlation between the HOMO energy and the long-term inhibition. All “cy” compounds except **cy**{*2,2*} scaled positively with the van der Waals interaction energy. Consistent with this, subsets of the compounds correlated negatively with the predicted density (atomic mass units/Å^3^) (Table S1). All compounds except **3c**{*1,1*}, **3b**{*2,2*}, **3a**{*2,2*} and DEET scaled positively with the fraction of rotable bonds. This suggests that the more flexible (and less dense) a compound, the more active, to the limit of **cy**{*1,1*} which was too small to fill the active space (see above). Consistent with this, the most active “cy” compounds, **cy**{*4,1*} and **cy**{*2,2*}, can access a large number of low energy rotamers, as seen in the potential energy surfaces (Fig. S5). Furthermore, all the “cy” compounds and active aromatics (**3c**{*1,1*}, **3a**{*2,2*} and **3b**{*2,2*}) scaled negatively with the polar accessible surface area. This suggests that the greater the accessible polar surface area the less active (Fig. 8C). Consistent with this, all the **cy**{*R_1_,1*} compounds scaled positively with logP_o/w_, suggesting that the more hydrophobic the more active.

Taken together, all our data suggest that long-term inhibition in the electrophysiological assay is a good predictor for alteration of host selection in the behavioral assay and that the active space delineated by long-term inhibition is also the active space for the alteration of the mite's host selection preference.

## Discussion

It is well known that the honey bees are chemically sensed by its obligatory parasite, *Varroa destructor*, presumably through olfactory sensilla located on its foreleg in a pit sensory organ (for a brief overview see [29]). In this study we were able to show for the first time that the *Varroa* sensory organ on the foreleg responds to honey bee headspace (kairomones) and that these responses can be measured by electrophysiology (EP). The *Varroa* EP response to honey bee volatiles consisted of a depolarization that was ∼30% larger than the response to air. This relatively modest response, compared to *e.g.* electroantennogram depolarizations of Lepidoptera to their sex pheromones, is probably due to the low abundance of olfactory sensilla on the foreleg, relative to mechanosensory sensilla. Nonetheless, the increase in the EP response to bee volatiles was dose dependent and sensitive enough to be used for screening of potential olfactory disrupting compounds. In this manuscript we investigated the possibility of disrupting the *Varroa* responses to honey bee volatiles by synthetic volatiles previously developed for gypsy moth olfactory disruption [19]. We tested two classes of compounds: dialkoxybenzenes and 5-2′hydroxyethyl-2-cyclopenten-1-ol ether derivatives (“cy” compounds). In particular, we have compared the effect of four dialkoxybenzenes that showed activity against insects [19],[30],[31], and six “cy” compounds on the ability of the *Varroa* olfactory organ to detect stimuli consisting of nurse honey bee volatiles. We utilized the headspace of nurse bees as a positive stimulus. Two activities were assessed in the EP assay: 1) decreased responses to honey bee headspace volatiles when the compound was given simultaneously (short-term inhibition) and 2) decreased responses to honey bee headspace volatiles puffed after a mixed compound/headspace stimulus (long-term inhibition). The effect varied depending on the compounds tested. The long-term olfactory inhibition fit well with our former behavioral observations that HCO-2-169, but not **3c{** *2*,*3*} (which was inactive), had a significant effect on *Varroa* host selection, eliminating significant nurse preference relative to untreated control [16]. In contrast, compound **3c**{*2,3*} (1-ethoxy-4-propoxybenzene) was the best long-term inhibitor on gypsy moth antennal responses to the pheromone [20].

To reveal structure activity relationship of this inhibitory effect we focused on HCO-2-169, a mixture of 5 racemic substituted cyclopentenes that differ in the length of the ether functional group. Among the five components of HCO-2-169, **cy**{*1,1*}, **cy**{*2,1*}, **cy**{*3,1*}, **cy**{*4,1*} and **cy**{*5,1*}, only **cy**{*1,1*} proved ineffective; the others showed various degrees of inhibitory effects short and/or long. One of the most effective compounds was **cy**{*4,1*}. This compound caused significant and dose-dependent inhibition of foreleg response to honey bee volatiles. Intriguingly, the long term effect was achieved at lower dose than a short term effect (0.01 µg and 0.1 µg, respectively). It should be indicated that, although these compounds were not expected to act by themselves on the olfactory system, **cy**{*4,1*} at 0.1 µg initially stimulated electrophysiological response in the *Varroa* sensory organ, but inhibited its subsequent response to honey bee volatiles. The absolute duration of such inhibition is impossible to determine on the detached organ whose function deteriorates within a short period of time, about half an hour. However for comparison the long term effect of **cy**{*4,1*} (0.1 µg) and **3b**{*2,2*} on *Varroa* foreleg lasted for over 60 s in contrast to the effect of **3c**{*2,3*} on gypsy moth antennae that decayed within 30 s [19]. The mechanism of such inhibition is unclear at this point.

The pit sensory organ (reviewed by Rosenkranz, [29]) consists of nine internal sensilla and nine long hair sensilla surrounding the organ. Some of the sensilla (at least six) are wall pore sensilla that bear similarity to the olfactory sensilla of other arthropods [25]. Despite great progress in recent years in the study of insect olfactory systems, not much information is available on the mechanisms behind olfactory signal transduction in mites. There are remarkable similarities in the general structure of olfactory systems in the animal kingdom [32],[33], but there are also differences. In insects the detection of volatiles is mediated by odorant binding proteins (OBPs), olfactory receptors (ORs) and the olfactory coreceptor (Orco) or variant ionotropic receptors (IRs), whereas in mites the mechanism is still unidentified. So far, ORs and Orco have not been reported in non-hexapods. It has been recently hypothesized by Viera and Rozas (2011) [38] that, unlike in Insecta (hexapoda), in Chelicerata ORs evolved from the gustatory receptor (GR) family. In contrast, IRs are present in all protostome species examined. One specific IR, IR25a, is conserved across protostomes and orthologs were reported in *Dapnia pulex* (Crustacea) and *Ixodes scapularis* (Arachnids) [37].

Theoretically, the EAG inhibiting compounds can interfere with any of the events prior to the activation of a sensory neuron. The first stage at which the compound could interfere is the interaction between the OBP and the natural odorant. Such interference has been proposed for dialkoxybenzenes that slow the EAG recovery rate in gypsy moths [20]. The next stage would be the interaction of the odorant with its respective receptor, along with ion channel opening or closing, and the last stage would be recovery of the system by the action of arrestin [34] and ion pumps [35]; reviewed by Plettner and Gries [19].

As electrophysiological studies are not enough to indicate if the chemical compounds are agonists or antagonists of the olfactory signal, the effect of the **cy**{*4,1*} compound was evaluated in a behavioral assay, in which *Varroa* was presented with a choice between nurse and forager. This assay clearly showed that inhibition of EP responses to nurse honey bee volatiles correlates with a profound behavioral change: a total reversal of the commonly observed [15],[10]],[36] nurse preference by the mite. An expected behavioral effect of a chemosensory disruptive compound would be a lack of preference, yet in this case the inhibiting compounds caused an inversion in preference. The mechanism of the reversal phenomenon is still enigma. As was already described, there is obviously an effect on a peripheral olfactory system, so it should be related to disruption of chemical recognition process. It has been indicated that *Varroa* discriminate between the hosts based on cuticular hydrocarbon profiles. The profile of nurses and foragers are known to be different [12],[26]. The difference relies on a large number of compounds and is mostly qualitative. Only putative forager-based repellents but no nurse-specific attractants have been described so far. We can speculate that since the recognition is based on a profile rather than on single compounds, the disrupting compound is causing conformational changes in the affinity of odorant biding proteins or the receptor, thereby inhibiting the sensing of some of the volatile/s. This inhibition leads to a change in the profile identified by the *Varroa* and accordingly to reversal of their preference. Identification of precise volatiles affected by the active disruptive compound is currently under study in our lab.

The specificity of these EP and behavioral effects of **cy**{*R_1_,1*} compounds needs to be explored further, by testing the individual enantiomers of these compounds. Furthermore, the active compounds need to be tested within the hive environment. Finally, to be developed into a possible anti-*Varroa* agent, the disrupting compounds should be also tested for their effect on bees. Due to specificity in volatile-receptor interactions and expected differences in ORs or IRs between insects and arachnids, there is a high probability that (a) *Varroa*-specific disrupting compound(s) can be identified.

The disruption of *Varroa* behavior by the compounds described above is not immediately lethal to the mites. However, we expect them to play a role in integrated *Varroa* management contributing to decrease in *Varroa* infestation via several mechanisms, e.g.: 1) forager infestation is expected to drive mites away from the brood, and 2) mites away of the brood cells are more exposed to the natural cleaning behavior of the bees and “soft” acaricides thus increasing the efficacy of the latter.

In conclusion, this study is the first to report a phenomenon of specific olfactory disruption in an arthropod other than an insect. A number of synthetic compounds that inhibit the electrophysiological response of the *Varroa* foreleg to nurse bee volatiles were identified. Two such inhibitors are *cis* 5-(2′-methoxyethyl)-cyclopent-2-en-1-butoxyl diether, **cy**{*4,1*}, and 1,3-diethoxybenzene, **3b**{*2,2*}. Their dose-dependent inhibition of the *Varroa* olfactory organ on the foreleg is mirrored in a significant behavior-modifying effect. The behavioral effect of **cy**{*4,1*} and **3b**{*2,2*} consists of the *Varroa* mites switching their host preference from nurse to forager. The superimposed, energy-minimized structures of these two compounds delineate an “active space.” Less active or inactive compounds either do not fit into that space or do not fill it sufficiently. The mode of action of these compounds, at the cellular level of the sensory organ, is to be resolved. Also the implementation of these compounds remains to be evaluated, along with the potential effects on honey bees. The disruption of *Varroa* communication is a promising step towards development of semiochemicals as a tool to control this major apicultural pest. The method developed in this and previous studies provide a tool for future screening of any potential olfactory inhibiting compounds.

## Supporting Information

Figure S1 ***Varroa*** **foreleg electrophysiological response amplitude.** Comparison between the responses to three sequential stimuli of five-bee headspace. ANOVA repeated measures followed by Tukey-Kramer post hoc tests. Bars marked by different letters are significantly different, F(2,12) = 0.0407, p = 0.96 (n = 7). (TIF)

Figure S2 **The effect of cy{** ***2,2*** **} on** ***Varroa*** **host choice between a nurse and a forager bee.** The compound was tested at different doses (0.01 µg, 0.1 µg, 10 µg) (OR = 54, (95% CI 15.3–231.9): data are the percentage of *Varroa* that selected a particular host 180 min from the beginning of the experiment in the presence of hexane (control) or disrupting compound. Numbers within the bars show the number of *Varroa* choosing each of the hosts. (TIF)

Figure S3 **Dose responses of long-term inhibitory compounds 3a{** ***2,2*** **}, 3c{** ***2,2*** **} and 3c{** ***1,1*** **}.** The responses of the *Varroa* forelegs to stimulation with different amounts of each compound and with the headspace from 5 nurse bees (normalized values against the response to air %, average +SE). ANOVA repeated measures followed by Tukey-Kramer post hoc tests. Bars marked by different letters are significantly different, *p*<0.05, (n = 6). (TIF)

Figure S4 **Overlays of structures. A.** Overlay of **cy**{*4,1*} and **cy**{*2,2*} (see discussion for details). **B**. Overlay of **cy**{*4,1*} and DEET. (TIF)

Figure S5 **Potential energy surfaces (PES) of cy{** ***4,1*** **} and cy{** ***2,2*** **}.** The potential energy surfaces represent the conformational energy for rotation around a pair of adjacent dihedral angles (*a1*–*a6*) shown with the structures of **cy**{*4,1*} and **cy**{*2,2*} at the top. The black dot on each graph indicates the energy minimum (node) of the structure after the double dihedral angle simulation. (TIF)

Table S1 **Correlations between activity and calculated molecular properties.**
(DOC)
