# Correction: Can We Disrupt the Sensing of Honey Bees by the Bee Parasite *Varroa destructor*?

**DOI:** 10.1371/journal.pone.0116127

**Published:** 2014-12-19

**Authors:** The PLOS ONE Staff

An affiliation for the first author is missing. Nurit Eliash is also affiliated with: Institute of Agroecology and Plant Health, Robert H. Smith Faculty of Agriculture, Food and Environment, Hebrew University of Jerusalem, Rehovot, Israel
