# Supplementary figures and images for: Can We Disrupt the Sensing of Honey Bees by the Bee Parasite Varroa destructor?
Source: PLoS One. 2014 Sep 16;9(9):e106889. doi: 10.1371/journal.pone.0106889 (PMC4167332; doi:10.1371/journal.pone.0106889)

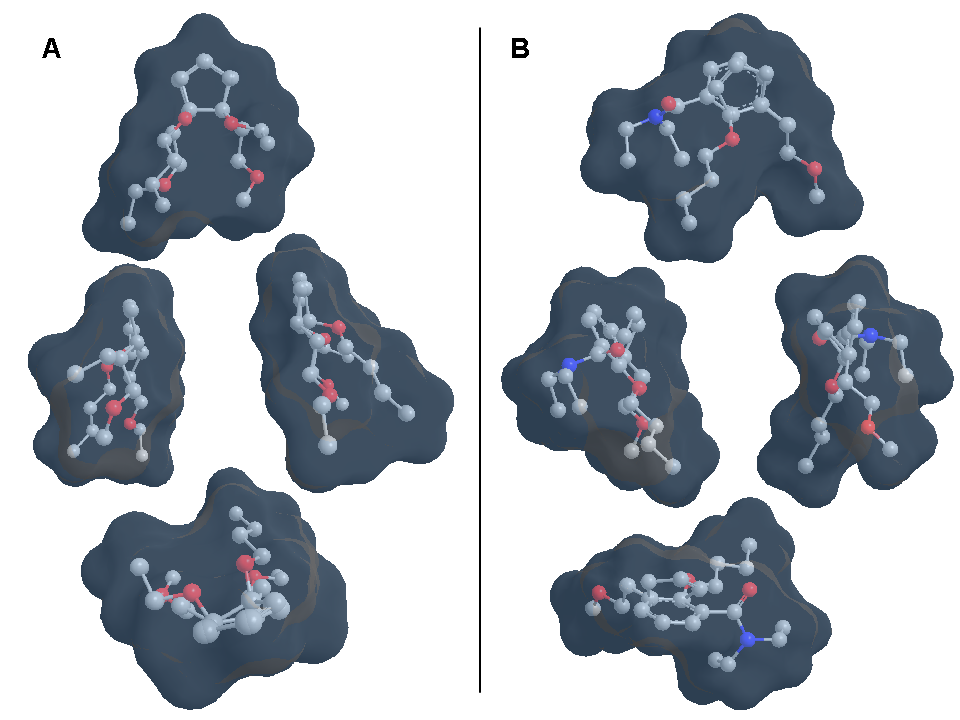

Supplement: Figure S4 — (TIF) [file pone.0106889.s004.tif]

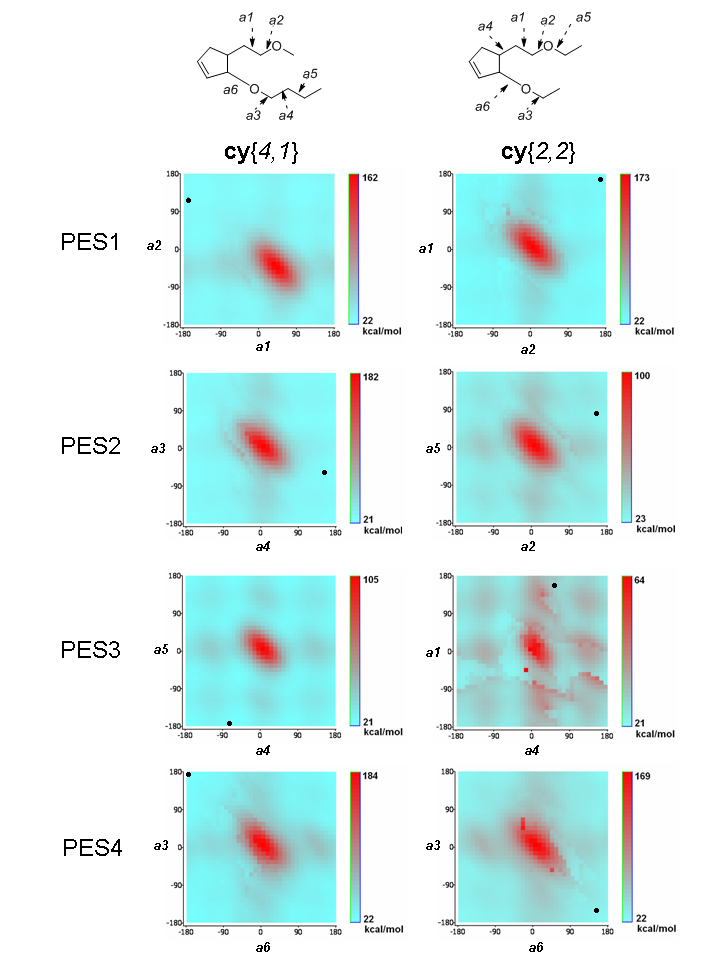

Supplement: Figure S5 — (TIF) [file pone.0106889.s005.tif]
