# Supplementary material for: Can We Disrupt the Sensing of Honey Bees by the Bee Parasite Varroa destructor?
Source: PLoS One. 2014 Sep 16;9(9):e106889. doi: 10.1371/journal.pone.0106889 (PMC4167332; doi:10.1371/journal.pone.0106889)
Supplement: Table S1 — (DOC) [file pone.0106889.s006.doc]

**Table S1.**  Correlations between activity and calculated molecular properties

| **Activity** ^a^ | **Calculated property** | **Compounds included** | **Line equation** | **R^2^** |
| --- | --- | --- | --- | --- |
| Δ STI (%) | HOMO (PM3) (eV) | Aromatic compounds | -0.01x - 8.63 | 0.580 |
|  | LUMO (PM3) (eV) | Aromatic compounds | -0.003x - 0.326 | 0.311 |
|  | LUMO-HOMO (PM3) (eV) | Aromatic compounds | 0.01x + 8.96 | 0.579 |
| Δ LTI (%) | HOMO (AM1) (eV) | **3c** compounds | -0.005x -8.496 | 0.909 |
|  | HOMO (PM3) (eV) | **3c** compounds | -0.006x - 8.609 | 0.776 |
|  | Van der Waals Interaction Energy (ErvdW) (kcal/mol) | All “cy” compounds except **cy**{*2,2*} | 2.5×10^5^x + 2.0×10^7^ | 0.839 |
|  | density (AMU/Å^3^) | **3c**{1,1}, **cy**{3,1}, **cy**{2,2} and **cy**{5,1} | -0.007x + 1.03 | 0.991 |
|  | “ | **3c**{1,3}, **3c**{2,2}, **cy**{2,1}, **cy**{4,1} | -0.004x + 0.937 | 0.946 |
|  | Fraction of rotable bonds (b_rotR) | All compounds except **3c**{*1,1*}, **3b**{*2,2*}, **3a**{*2,2*} and DEET | 0.007x + 0.344 | 0.788 |
|  | Polar accessible surface area (ASA_P) (Å^2^) | All compounds except **3c**{*2,3*}, **3c**{*2,2*}, **3c**{*1,3*} and DEET | -1.4x + 104 | 0.927 |
|  | Accessible surface area (ASA) (Å^2^) | All compounds except **3c**{*1,1*}, **3b**{*2,2*}, **3a**{*2,2*}, **cy**{*2,1*} and DEET | 3.4x + 403 | 0.691 |
|  | logP_o/w_ | All **cy** compounds | 0.06x + 1.26 | 0.691 |

^a^ Taken from the data in Figure 3.
